# Depressive Mood Assessment Method Based on Emotion Level Derived from Voice: Comparison of Voice Features of Individuals with Major Depressive Disorders and Healthy Controls

**DOI:** 10.3390/ijerph18105435

**Published:** 2021-05-19

**Authors:** Shuji Shinohara, Mitsuteru Nakamura, Yasuhiro Omiya, Masakazu Higuchi, Naoki Hagiwara, Shunji Mitsuyoshi, Hiroyuki Toda, Taku Saito, Masaaki Tanichi, Aihide Yoshino, Shinichi Tokuno

**Affiliations:** 1 Department of Bioengineering, Graduate School of Engineering, The University of Tokyo, Tokyo 113-8656, Japan; nakamura@bioeng.t.u-tokyo.ac.jp (M.N.); higuchi@bioeng.t.u-tokyo.ac.jp (M.H.); mitsuyoshi@bioeng.t.u-tokyo.ac.jp (S.M.); tokuno@bioeng.t.u-tokyo.ac.jp (S.T.); 2 PST Inc., Yokohama 231-0023, Japan; omiya@medical-pst.com; 3 AGI Inc., Tokyo 113-8655, Japan; hagiwara@agi-web.co.jp; 4 Department of Psychiatry, National Defense Medical College, Tokorozawa 359-8513, Japan; toda1973@gmail.com (H.T.); t.saito3025@gmail.com (T.S.); mtanichi@gmail.com (M.T.); aihide@ndmc.ac.jp (A.Y.)

**Keywords:** mood disorder assessment, vitality, mental activity, voice index, emotion analysis, noninvasiveness

## Abstract

Background: In many developed countries, mood disorders have become problematic, and the economic loss due to treatment costs and interference with work is immeasurable. Therefore, a simple technique to determine individuals’ depressive state and stress level is desired. Methods: We developed a method to assess specific the psychological issues of individuals with major depressive disorders using emotional components contained in their voice. We propose two indices: vitality, a short-term index, and mental activity, a long-term index capturing trends in vitality. To evaluate our method, we used the voices of healthy individuals (*n* = 14) and patients with major depression (*n* = 30). The patients were also assessed by specialists using the Hamilton Rating Scale for Depression (HAM-D). Results: A significant negative correlation existed between the vitality extracted from the voices and HAM-D scores (r = −0.33, *p* < 0.05). Furthermore, we could discriminate the voice data of healthy individuals and patients with depression with a high accuracy using the vitality indicator (*p* = 0.0085, area under the curve of the receiver operating characteristic curve = 0.76).

## 1. Introduction

Mood disorders have become a major issue in several developed countries [1], and the economic loss due to treatment costs and interference with work is enormous [2]. Therefore, a simple technique to determine individuals’ depressive state and stress level is desired. Self-administered psychological tests, such as the General Health Questionnaire (GHQ) [3,4] and Beck Depression Inventory (BDI) [5,6], can be used as screening methods for patients with major depressive disorders. In addition, a stress-check method that uses biomarkers in saliva [7] and blood has been proposed [8]. Although self-administered psychological tests are useful for early detection and as diagnostic aids, there is a problem with reporting bias in which specific information such as smoking history and medical history are selectively suppressed or expressed by participants [9]. Stress-check methods that use biomarkers also have problems such as the cost of the test and the burden on the participants during specimen collection, i.e., they are not convenient. On the other hand, with the recent widespread use of smartphones, pathological analysis using voice data has become popular [10,11,12]. Voice analysis using smartphones is not only noninvasive, but also, it does not require a dedicated device; thus, it can be performed conveniently and remotely.

The voice of a depressed person has dull, monotonous, and lifeless [13] features, and listeners can perceive patients’ prosody [14,15]. The relationship between mood illness and voice has been observed in previous studies, e.g., studies regarding the speaking rate of patients with depression [16,17,18] and studies on switching pause and percent pause of patients with depression [15,18]. Vicsi et al. used frequency analysis and showed that the shimmer and jitter in vowels as voiced by patients with depression were higher than those of healthy people, and the first and second formant frequencies were low [19]. Low formant frequencies for the same utterance mean that the voice is low.

The mel-frequency cepstral coefficient (MFCC) is often used for voice recognition. Taguchi et al. [20] showed that MFCC2 (the second dimension of MFCC) is effective in classifying patients with depression and individuals without depression; however, MFCC2 did not correlate with the severity of depression measured by the Quick Inventory of Depressive Symptomatology—Self-Report, Japanese version (QIDS-SRJ) [21]. Wang et al. showed that loudness, MFCC5 and MFCC7 are effective indicators that could be utilized for identifying depression [22].

Research on voice emotion recognition and measurement of emotional arousal level using voice have been encouraged. For example, the relationship between arousal level and voice intensity or pitch has been documented [23,24]. Stress is known to have an impact on emotions [25], and a method is being developed to estimate stress through emotion instead of analyzing stress directly from voice data [26,27,28]. Mitsuyoshi et al. [26] proposed an algorithm that estimates the expression of emotion from emotion components of the voice—the vocal affect display. In addition, they experimentally analyzed the relationship between this index and stress and estimated individuals’ stress level from their voice.

We are focusing on major depression-like depressive symptoms associated with stress accumulation, which has become a problem in industry in recent years. Since emotions such as sadness are amplified in the process of completion of stress-induced depressive symptoms, we thought that the progress of depressive symptoms could be detected by interposing emotion measurement. In the present study, sensibility technology (ST) that analyzes emotion in speakers’ voices was used [29,30,31]. More specifically, this study proposes a method to assess the mood disorder of a speaker from the emotional components in their voice using ST, with a focus on the relationship between mood disorder and emotions.

## 2. Materials and Methods

### 2.1. Acquisition of Voices

In this study, we collected voice data in two categories—healthy individuals and outpatients with depression. All participants provided written consent. Voice acquisition of the patient group was performed intermittently from August 2013 to October 2014 with outpatients at Kitahara rehabilitation hospital in Japan. Voices were recorded during patients’ conversations with physicians during examination. All data were then confirmed audibly; overlaps with other speakers and background noises were removed manually.

Voices of healthy people were acquired from February to mid-May 2015. During the acquisition period, participants worked normally at their jobs without visiting medical facilities for a mood illness. Voice acquisition was continuously performed once every several days; each time, 14 types of fixed phrases were read aloud twice. Voices were recorded in a quiet environment with little background noise.

Voices were recorded by a gun microphone (AT9944: Audio-Technica, Tokyo, Japan) placed approximately 100 cm from the participant or by a pin microphone (ME52W: OLYMPUS, Tokyo, Japan) attached to the chest at approximately 15 cm from the participant’s mouth. The recording device was MS-PST1 (NORITSU KOKI, Wakayama, Japan) which was not commercially available.

Table 1 shows participants’ information per group. It should be noted that the number of participants and the number of data differed because data may have been collected multiple times from the same participant on different days. The average number of data collected per healthy person was 24.4 ± 33.3 for men and 6.3 ± 6.1 for women. For patients with depression, it was 6.0 ± 2.9 for men and 6.8 ± 3.2 for women. These collected data were used to create algorithms to calculate vitality and mental activity.

Regarding the above-described recorded voice, a healthy person’s voice was recorded uttering a fixed phrase. On the other hand, a patient’s voice was recorded during free speech in the form of a dialogue with a doctor, and the type of speech differed between a healthy person and a patient. Furthermore, the recording location differed. To unify both speech types and recording environments, data for algorithm verification were collected at the National Defense Medical College Hospital in Japan with participants’ consent.

First, from December 2015 to June 2016, fixed-phrase reading voices were collected from outpatients with major depression. Table 2 shows the 17 types of Japanese fixed phrases that were used for recording. At the time of voice collection, specialists evaluated patients’ depression severity using the Hamilton Rating Scale for Depression (HAM-D) [32]. The HAM-D is not a self-assessment-type psychological test; rather, experts, such as doctors, evaluate the characteristic items of depression symptoms. The purpose of the HAM-D is for a professional to objectively quantify an individual’s depressive state. On the other hand, for voices of healthy individuals, in mid-December 2016, the same fixed phrase reading voices as the patients were recorded in the same examination room as the patients. However, for healthy people, severity assessment using the HAM-D was not conducted.

Patients were included if they had been diagnosed with a major depressive disorder according to the Diagnostic and Statistical Manual of Mental Disorders [33] and were aged over 20 years. They were excluded if they had been diagnosed with a serious physical disorder or organic brain disease. They were diagnosed by a psychiatrist using the Mini-International Neuropsychiatric Interview [34]. The attending physician explained to the patients the purpose and content of the study, that the anonymity and confidentiality of their data were guaranteed, that they were free to withdraw at any time and that there was no disadvantage if they refused to complete the study. Additionally, only when consent was obtained, the attending physician conducted voice recording after normal medical treatment. Participants were not rewarded for their participation.

The protocol of this study was designed in accordance with the Declaration of Helsinki and relevant domestic guidelines issued by the concerned authority in Japan. The protocol was approved by the ethics committee of the National Defense Medical College (No. 2248) and the Kitahara Rehabilitation Hospital Ethics Committee (No. 3). According to Japanese law, the sensitivity of audio files is similar to that of any other personal information and cannot be published without consent. In this research protocol, we did not obtain consent from the subjects to publish the raw audio files as a corpus.

These voices were recorded by a pin microphone, ME52W (OLYMPUS, Tokyo, Japan), attached to the chest approximately 15 cm from each participant’s mouth. The recording device used was an R-26 Portable Recorder (Roland, Shizuoka, Japan). Table 3 shows participants’ information for algorithm verification. The number of healthy individuals for verification and the number of their voice data were the same because they were collected only once from each healthy participant. Regarding patients, some participants performed multiple data acquisitions. Seven, three, and one performed data acquisition twice, thrice, and four times, respectively. Data were acquired only once from the remaining 19 people. The recording format of the voices was linear PCM, the sampling frequency was 11,025 Hz, and the number of quantization bits was 16 bits.

### 2.2. Voice Emotion Analysis System

We used the software ST Ver. 3.0 (AGI Inc., Tokyo, Japan) [29,30,31] to extract emotions from participants’ voices. The categories of emotional elements detected by ST software are “anger”, “joy”, “sorrow”, “calmness” and “excitement”. The strength of each emotion is represented as an integer value from “0” to 10. A value of “0” means that the input speech does not contain the emotion at all. A value of 10 means that the input speech contains the emotion most strongly. The unit of speech emotion analysis by ST software is “utterance”. This is a part of continuous voice divided by breath. When a silent state changes to a speech state, it is considered that an utterance has started. When the speech state continues for a certain period and changes to silence, it is considered that the utterance has ended. The presence of the silent state or speech state is determined from the volume using a threshold. The threshold was adjusted manually for each recording, as the volume of the audio is affected by the participant and the condition of the recording.

### 2.3. Algorithm

#### 2.3.1. Vitality and Mental Activity

We proposed two scales—vitality and mental activity—as indices for the degree of mood disorder obtained through voice analysis. Generally, “vitality” can be defined in diverse ways; however, here, vitality refers to a scale that measures low for patients with illnesses such as depression and high for healthy people. The main difference between vitality and mental activity is the duration of the measurement. Vitality is calculated from the emotional components of the voice (calmness, anger, joy, sorrow and excitement) based on short-term voice data, such as a single phone conversation or a hospital visit.

On the other hand, mental activity is calculated based on vitality data accumulated over a certain period. Vitality changes based on the conditions at the time of measurement in the same manner that blood pressure changes between post-workout and rest. Similar to accurate identification of high blood pressure through long-term monitoring, in this study, we aimed to accurately assess mood disorders by introducing mental activity.

#### 2.3.2. Vivacity and Relaxation

To calculate vitality, we introduced two new indices: “relaxation” and “vivacity”. To define these indices, we used four out of five indices output by ST: calmness, joy, sorrow, and excitement.

The fifth edition of the Diagnostic and Statistical Manual of Mental Disorders describes the characteristics of a major depressive episode as a continuing depressive state with loss of interests and happiness and feelings of sorrow and emptiness [33]. In contrast, if there is a component of joy more prevalent, relative to sorrow in emotion, it is considered a good mood state. Consequently, vivacity for an utterance was defined as follows:

(1) $$vivacity=\frac{joy}{joy+sorrow}$$

Stress and tension are major factors in mood disorders. On the other hand, the relaxed state is mentally positive; thus, relaxation for an utterance was defined as follows:

(2) $$relaxation=\frac{calm}{calm+excitement}$$

In other words, relaxation increases with the increasing calmness component of emotion and decreasing excitement. Each emotional value output by ST is expressed with integers in the range of 0–10. Therefore, vivacity and relaxation become real numbers in the range of 0.0–1.0. Vivacity and relaxation, as defined above, were calculated for each utterance. Furthermore, we define vivacity and relaxation for an acquired voice as the mean value for each utterance contained in the acquired voice.

#### 2.3.3. Vitality Calculation Algorithm

**Figure 1:**
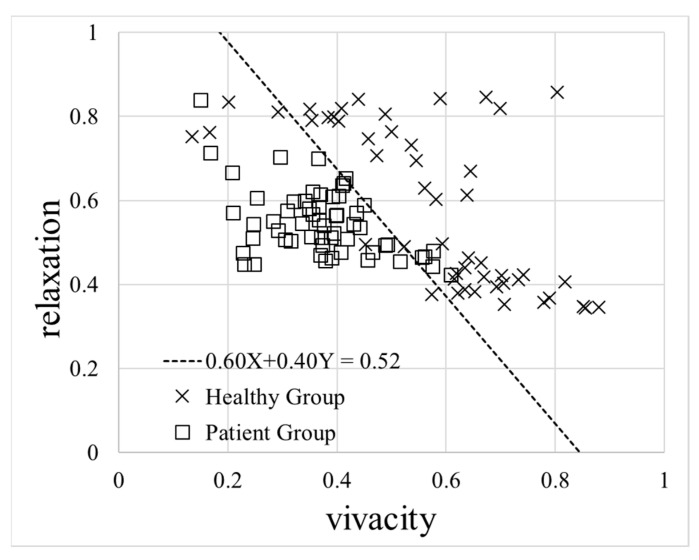
Scatter plot of relaxation and vivacity. Data are plotted for each voice acquisition. There are 50 data for the 13 people in the healthy group and 58 for the 9 people in the patient group. The straight line separates the healthy group from the patient group (0.60X + 0.40Y = 0.52).

Vitality was calculated as the weighted mean of vivacity and relaxation defined in the previous section. Figure 1 shows a scatter plot of relaxation and vivacity as calculated from the data for algorithm preparation. Based on the straight line in the figure, vitality for each acquired voice was defined as follows:

(3) vitality=0.60×vivacity+0.40×relaxation

#### 2.3.4. Mental Activity Calculation Algorithm

Vitality was calculated from short-term voice data such as a single examination or consultation. Therefore, depending on participants’ current mood, even healthy people might score low in vitality, while patients may score high. To compensate for such a weakness, mental activity was calculated from long-term trends in vitality. Specifically, to express long-term trends in vitality, we calculated the mean of accumulated vitality ($\bar{vitality}$).

Furthermore, when vitality has little fluctuation and is stagnant at low values, the patient was determined to have low mental activity. To actualize such a determination, we introduce a new index: standard deviation (vitalitySD) that expresses variations in vitalities for utterances contained in acquired voice. Then, the mean of vitality standard deviation of the accumulated acquired voice ($\bar{vitalitySD}$) was calculated.

**Figure 2:**
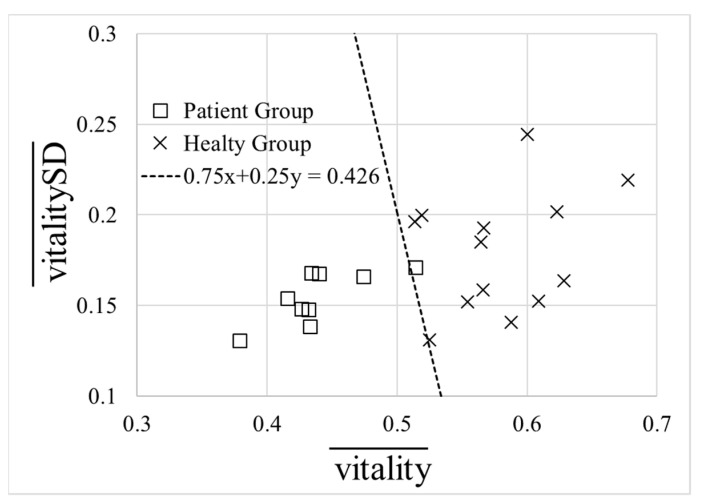
Scatter plot of mean vitality and the mean standard deviation of vitality for each participant (N = 22).

Figure 2 is a scatter plot of the mean vitality and mean standard deviation of vitality for each participant calculated from the data for algorithm preparation. The number of data plotted was 13 people for the healthy group and 9 people for the patient group (*n* = 22). When calculating the mean, we used all acquired voice data. In the figure, we added a straight line that separates the healthy group and the patient group (0.75X + 0.25Y = 0.426). Based on this line and using the mean and standard deviation of vitality, we define mental activity as follows:

(4) $$mental\_activity=0.75\times \bar{vitality}+0.25\times \bar{vitalitySD}$$

### 2.4. Analysis Method

According to the definition of Zimmerman et al. [35], the data of the patient group were divided into two groups by HAM-D score: no depression (≤7) and depression (≥8). The vitality of the three groups (i.e., these two and the healthy group) were compared with each other.

P-values from Tukey–Kramer tests, the area under the curve (AUC), sensitivity, and specificity were used to evaluate the classification accuracy of vitality. Furthermore, the power of the test and effect size using Cohen’s d were calculated. For all analyses, statistical significance was set at *p* < 0.05.

The following analysis was performed using the statistical software R, version 3.6.1 (2019-07-05) [36], unless otherwise specified. We used the R packages of Epi version 2.41 for AUC calculation, multcomp version 1.4.16 for the Tukey–Kramer test, effsize version 0.8.1 for Cohen’s d and pwr version 1.3.0 for sample size estimation. The operating system used was Windows 10 (Microsoft Corp., Redmond, WA, USA).

## 3. Results

### 3.1. HAM-D Score

The mean values of HAM-D score in each group are shown in Table 4. The number of participants in each group was 11 men and 8 women in the no depression group, and 8 men and 3 women in the depression group.

### 3.2. Performance Evaluation of Vitality

**Figure 3:**
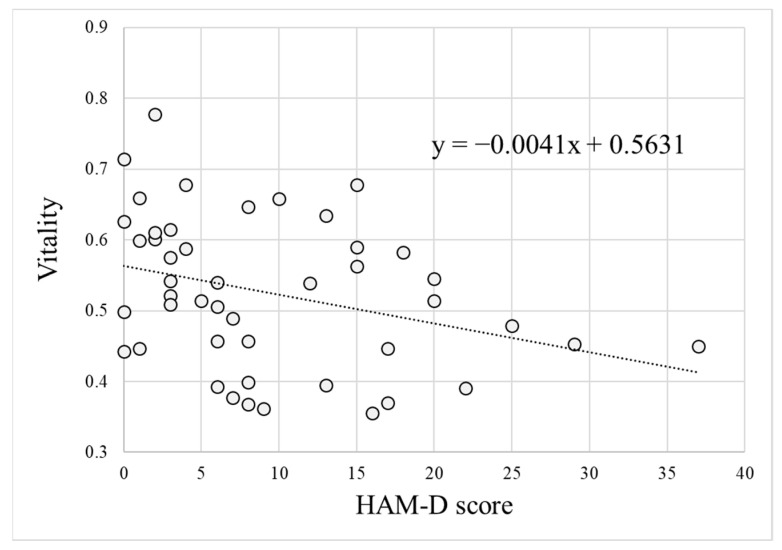
Relationship between Hamilton Rating Scale for Depression (HAM-D) score and vitality in the data of patient group for algorithm verification. The figure also shows the regression line for the data (y = −0.0041x + 0.5361).

We evaluated the performance of vitality using the data for algorithm verification, as shown in Table 3. Figure 3 shows the relationship between HAM-D score and vitality for 46 data obtained from the patient group. There was a significant negative correlation between the two (r = −0.33, *n* = 46, *p* < 0.05).

**Figure 4:**
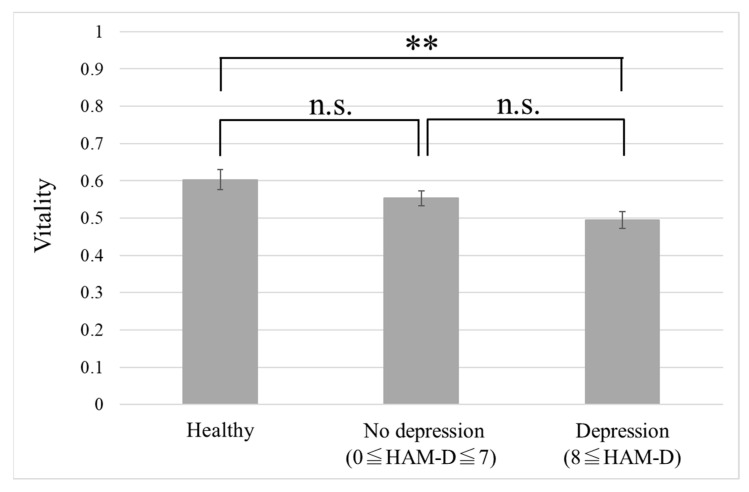
Comparison of vitality for each group. Error bars represent standard error. ** *p* < 0.01, n.s.: not significant. HAM-D: Hamilton Rating Scale for Depression.

Figure 4 shows the comparison of vitality scores of the healthy group, no depression group, and depression group. The mean vitality in each group was 0.60 ± 0.027 (*n* = 14), 0.55 ± 0.020 (*n* = 24), and 0.49 ± 0.022 (*n* = 22), respectively. The Tukey–Kramer test revealed significant differences between the healthy group and the depression group (*p* = 0.0085). The effect size of the vitality between the healthy and depression groups was 1.03. When this value was used as the effect size, with a significance level of 0.05, the power of the test was 0.84; this value is greater than 0.8, thereby indicating that the power of the test is large. However, when the number of data for the depression group was set to 22 and the power of the test was set to 0.8 with a significance level of 0.05 and effect size of 1.03, the required number of samples for the healthy group was 12.13. The number of data for the healthy group in this study is 14, so the actual data are slightly higher than the calculated requirement.

**Figure 5:**
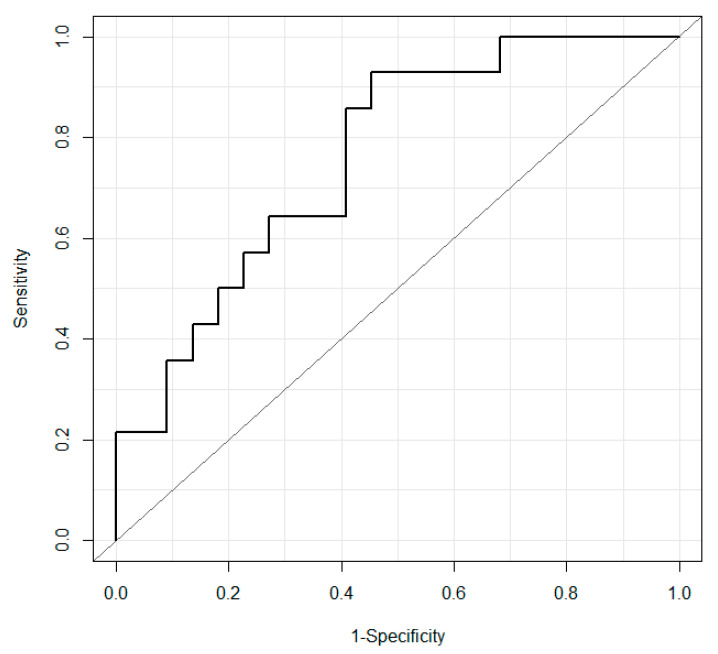
Receiver operating characteristic curves when using vitality to identify groups. The straight line represents y = x.

Next, to evaluate the discrimination performance of vitality, the AUC of the receiver operating characteristic (ROC) curve, sensitivity, and specificity were used. Figure 5 shows the ROC curves when vitality was used to identify whether the data for verification are for the healthy group or for the depression group. Here, the horizontal axis represents 1-specificity (false positive rate), and the vertical axis represents sensitivity (positive rate).

The AUC was 0.76, and the sensitivity and specificity were 0.93 and 0.55, respectively, regarding the discrimination performance between the healthy group and the depression group.

## 4. Discussion

In this study, we developed a method to measure mood disorders using emotional components contained in voice. Two indicators were proposed: vitality based on short-term voice data and mental activity calculated from long-term voice data. As shown in Figure 3, there was a significant negative correlation between vitality and HAM-D score (i.e., depression severity assessed by a physician). In addition, as shown in Figure 4 and Figure 5, we could discriminate the voice data of healthy individuals and patients with depression with a high accuracy using the vitality indicator. On the other hand, there was no significant difference between the healthy group and the no depression group with almost no depressive symptoms, even if they were outpatients with depression. This suggests the possibility of measuring treatment effects by vitality (i.e., voice).

In our previous study, we verified vitality with Romanian and Russian native speakers [37]. In this verification, BDI tests were conducted simultaneously with voice recordings. There was a significant difference between the mean vitality of the high-risk depression group (BDI scores ≥ 17) and the mean vitality of the low-risk depression group (BDI scores < 17; *p* < 0.05). Specifically, the scores for question 9—concerning suicidal ideation—took a value that ranged 0–3. There was a significant difference between the mean vitality of the low-risk suicide group (0 or 1 points) and the mean vitality of the high-risk suicide group (2 or 3 points; *p* < 0.01). In the future, we will examine the vitality of native speakers of other languages, such as English.

As a limitation of this research, only the fixed-phrase read-out speech was used for verification. To apply vitality to free speech such as a call, further verification is required. Furthermore, in the verification data, the number of voices collected for each participant, sex ratio, and age were not unified between the groups. These differences may be reflected in the features of voice.

Furthermore, mental activity was not validated because continuous data could not be collected sufficiently for the same participants in both the healthy group and the patient group. However, comparing Figure 1 and Figure 2 showing data for algorithm preparation, there is a possibility that mental activity can more accurately identify the data as compared to vitality, which will be addressed in the future.

Vitality and mental activity can be measured only by voice, and their advantages are that they are non-invasive and less expensive than self-administered tests such as the GHQ-30 and BDI and stress-check methods using saliva and blood. Moreover, it is also possible to record day-to-day state changes easily by implementing them on smartphones or other similar devices.

We developed a smartphone application that implements the algorithm for vitality and mental activity—called the Mind Monitoring System (MIMOSYS). We are currently conducting worldwide demonstration experiments using the MIMOSYS [38,39]. In the future, we plan to verify the effectiveness of vitality and mental activity with such large-scale data.

## 5. Conclusions

In this study, we developed a method to measure mood disorders from voice. The MIMOSYS implemented an algorithm for vitality and mental activity, which is a cost-effective and convenient measurement device. If the correlation between HAM-D score and vitality can be further enhanced, it may be used to aid doctors’ diagnoses in the future. By daily monitoring of vitality and mental activity using the MIMOSYS, we can encourage hospital visits for people before they become depressed or during the early stages of depression. This may lead to reduced economic loss caused by treatment costs and interference with work.

## Figures and Tables

**Table 1:** Experimental participant information for algorithm preparation.

Group	Sex	Number of Participants	Mean Age	Number of Data
Healthy	Male	9	42.9 ± 5.6	25
	Female	4	33.3 ± 15.4	25
Major depression	Male	4	54.0 ± 12.0	24
	Female	5	49.4 ± 15.4	34

**Table 2:** Seventeen phrases used for recording.

No.	Phrase in Japanese	Purpose (Meaning)
1	I-ro-ha-ni-ho-he-to	Non-emotional (no meaning, like “a-b-c”)
2	Honjitsu ha seiten nari	Non-emotional (It is fine today)
3	Tsurezurenaru mama ni	Non-emotional (Having nothing to do)
4	Wagahai ha neko dearu	Non-emotional (I am a cat)
5	Mukashi aru tokoro ni	Non-emotional (Once upon a time, there lived)
6	a-i-u-e-o	Check pronunciation of vowel sounds (no meaning like “a-b-c”)
7	Ga-gi-gu-ge-go	Check sonant pronunciation (no meaning, like “a-b-c”)
8	Ra-ri-ru-re-ro	Check liquid sound pronunciation (no meaning, like “a-b-c”)
9	Pa-pi-pu-pe-po	Check p-sound pronunciation (no meaning, like “a-b-c”)
10	Omoeba tooku he kita monda	Non-emotional (While thinking, I have come far)
11	Garapagosu shotou	Check pronunciation (Galápagos Islands)
12	Tsukarete guttari shiteimasu.	Emotional (I am tired/dead tired)
13	Totemo genki desu	Emotional (I am very cheerful)
14	Kinou ha yoku nemuremashita	Emotional (I was able to sleep well yesterday)
15	Shokuyoku ga arimasu	Emotional (I have an appetite)
16	Okorippoi desu	Emotional (I am irritable)
17	Kokoroga odayaka desu	Emotional (My heart is calm)

**Table 3:** Experimental participant information for algorithm verification.

Group	Sex	Number of Participants	Mean Age	Number of Data
Healthy	Male	10	42.7 ± 6.0	10
	Female	4	35.0 ± 14.4	4
Major depression	Male	19	43.7 ± 11.0	34
	Female	11	53.9 ± 8.2	12

**Table 4:** Average value of Hamilton Rating Scale for Depression (HAM-D) score for each group.

Group	Number of Participants	Number of Data	Mean HAM-D Score ± SD
No depression (HAM-D ≤ 7)	19	24	3.1 ± 2.3
Depression (HAM-D ≥ 8)	11	22	16.1 ± 7.4

HAM-D: Hamilton Rating Scale for Depression; SD: Standard Deviation.

## Data Availability

According to Japanese law, the sensitivity of audio files is similar to that of any other personal information and cannot be published without consent. In this research protocol, we did not obtain consent from the participants to publish the raw audio files as a corpus. The datasets used and/or analyzed during the current study are available from the corresponding author upon reasonable request.
